# Charge-Domain Type 2.2 µm BSI Global Shutter Pixel with Dual-Depth DTI Produced by Thick-Film Epitaxial Process †

**DOI:** 10.3390/s25226997

**Published:** 2025-11-16

**Authors:** Toshifumi Yokoyama, Masafumi Tsutsui, Yoshiaki Nishi, Yoshihiro Noguchi, Masahiko Takeuchi, Masahiro Oda, Fenigstein Amos

**Affiliations:** 1 Tower Partners Semiconductor Co., Ltd., 800 Higashiyama, Uozu City 937-8585, Toyama, Japan; tsutsui.masafumi@tpsemico.com (M.T.); nishi.yoshiaki@tpsemico.com (Y.N.); noguchi.yoshihiro@tpsemico.com (Y.N.); takeuchi.masahiko@tpsemico.com (M.T.); oda.masahiro@tpsemico.com (M.O.); 2 Tower Semiconductors, Migdal Haemeq 23105, Israel; amosfe@towersemi.com

**Keywords:** CMOS image sensor, global shutter, charge domain, parasitic light sensitivity, dark current, modulation transfer function

## Abstract

We developed a 2.2 µm backside-illuminated (BSI) global shutter (GS) pixel featuring true charge-domain-correlated double sampling (CDS). To enhance the inverse parasitic light sensitivity (1/PLS), we implemented a thick-film epitaxial process incorporating a dual-depth deep trench isolation (DTI) structure. The thickness of the epitaxial substrate was 8.5 µm. This structure was designed using optical simulation. By using a thick epitaxial substrate, it is possible to reduce the amount of light that reaches the memory node. The dual-depth DTI design, with a shallower trench on the readout side, enables efficient signal transfer from the photodiode (PD) to the memory node. To achieve this structure, we developed a process for thick epitaxial substrate, and the dual-depth DTI can be fabricated with a single mask. This pixel represents the smallest charge-domain GS pixel developed to date. Despite its compact size, it achieves a high quantum efficiency (QE) of 83% (monochrome sample: wavelength = 560 nm) and a 1/PLS exceeding 10,000 (white halogen lamp with IR-cut filter). The pixel retains 80% of its peak QE at ±15° incident angles and maintains stable 1/PLS performance even under low F-number (F#) conditions.

## 1. Introduction

The demand for small pixel-size global shutter (GS) image sensors, capable of capturing distortion-free images of fast-moving objects, continues to grow. This functionality is particularly desirable for applications such as machine vision and high-end movie camera. Previously, we demonstrated a 2.5 µm front-side illuminated (FSI) GS pixel employing a light pipe structure [1]. However, in front-side illuminated FSI structures, as pixel size decreases, the proportion of the wiring area increases, resulting in a reduced optical aperture. In contrast, backside-illuminated (BSI) architectures are well-suited for enlarging the optical aperture, achieving higher quantum efficiency (QE) and providing greater flexibility in wiring design. As a result, the development of BSI GS pixels is actively progressing [2,3]. Another critical requirement for small pixels and low photon counts is noise suppression. Charge-domain pixels are particularly well-suited for minimizing pixel-level noise, making them highly advantageous for visible light imaging applications [4,5,6]. However, BSI introduces challenges in suppressing parasitic light sensitivity (PLS). For a high-performance GS sensor, the target 1/PLS value should be around 10,000 or higher. In addition, PLS needs to be suppressed even when the F# becomes small. Furthermore, even if the pixel size becomes smaller, a wide angular response is required. In this paper, we present a 2.2 µm charge-domain BSI GS pixel with best-in-class performance, achieved through an especially deep dual-depth DTI structure and a thick-epi process [7].

## 2. Device Structure

**Figure 1:**
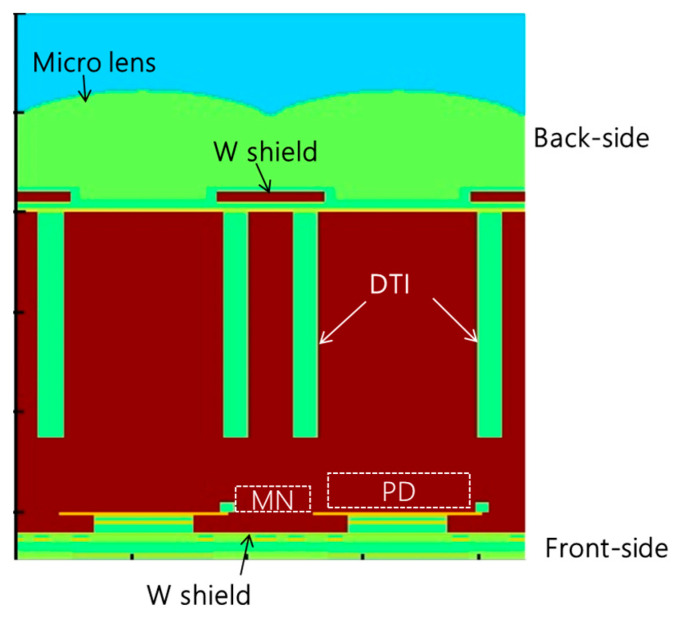
Cross-section of the BSI_GS sensor.

Figure 1 illustrates a basic cross-section of the charge-domain type BSI GS pixel [8]. The BSI_GS sensor in this report was fabricated using an n-type epitaxial substrate using a 65 nm process. Incident light is focused by a micro-lens and directed toward the photo diode (PD) region, which is surrounded by deep trench isolation (DTI). A fully buried memory node (MN) is located near the front-side surface to store charges until readout. This buried MN structure has been shown to effectively suppress dark current, as previously reported [9].

PLS arises when photoelectrons are generated within the MN or when electrons generated elsewhere in the pixel are inadvertently collected by the MN. This parasitic signal can lead to image artifacts during GS operation, particularly in highly dynamic scenes. PLS from photoelectrons generated outside the MN can be effectively mitigated through device design that ensures the PD collects all generated photoelectrons. In BSI pixels, tungsten shield (WS) is implemented on both the front and back sides [10]. The WS on the backside prevents direct incidence of light into the MN. On the other hand, the WS on the front side prevents reflected light from the wiring layer from entering the MN. Light penetration into the MN is further minimized by DTI.

**Figure 2:**
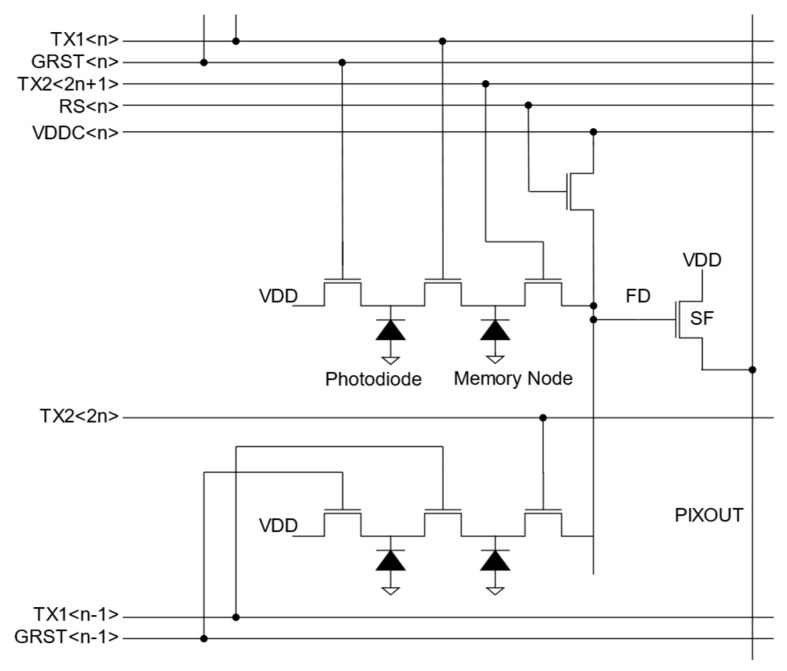
Pixel circuit schematic of GS pixel.

Figure 2 illustrates the circuit schematic of the developed GS pixel. To maximize the PD area, the row-select transistor was eliminated, and the floating diffusion (FD) was shared between two pixels [11,12]. Additionally, a narrow MN was designed to balance angular response and full-well capacity.

## 3. Development of 2.2 µm BSI Charge-Domain Global Shutter Pixel

### 3.1. Optical and Pixel Design

**Figure 3:**
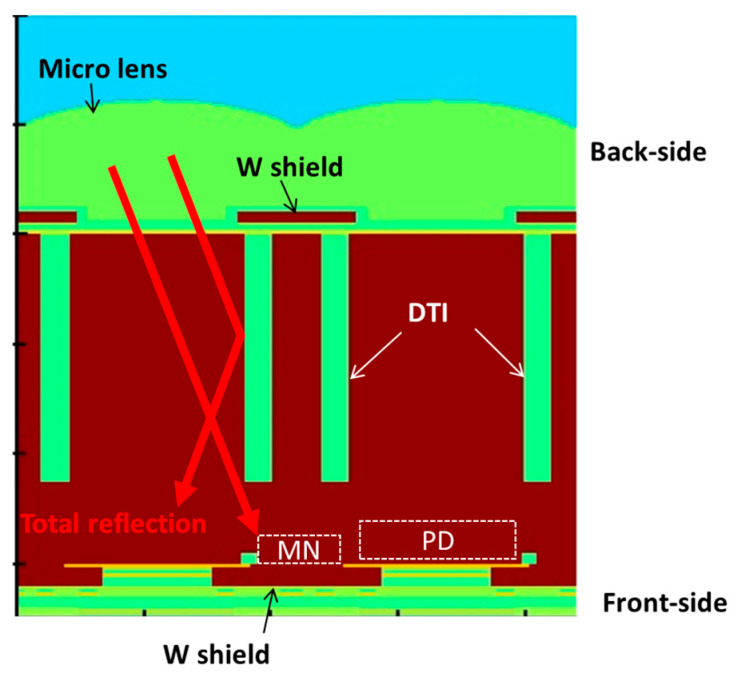
Effects of DTI structure and points for improvement.

The following section describes the optical design, beginning with the role of DTI. Since the DTI is filled with a low-refractive-index material, such as an oxide film, the oblique light inside the Si is totally reflected by the DTI as shown in Figure 3 [13]. This reflection significantly reduces the light penetration into the MN. However, beneath the bottom of the DTI, the light within the PD region is not reflected and can propagate into the MN. Therefore, increasing the depth of the DTI is essential for effective suppression of PLS.

**Figure 4:**
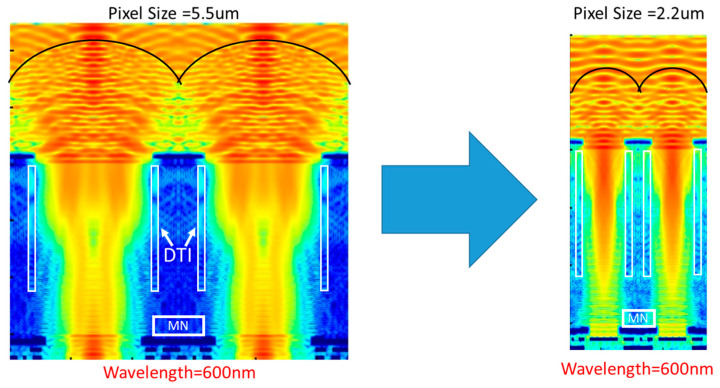
Impact of pixel size reduction.

Next, we describe the impact of pixel size reduction. Figure 4 compares the electric field distributions for pixel sizes of 5.5 µm and 2.2 µm based on the results of optical simulations. The simulation was carried out at a wavelength of 600 nm. In both cases, the epitaxial layer thickness was set to 6 µm. When the pixel size is 5.5 µm, the distance between the focal point of incident light and the MN is relatively large, resulting in minimal light penetration into the MN. In contrast, with a pixel size of 2.2 µm, the focal point is positioned closer to the MN, allowing more light to reach and penetrate it. Consequently, as pixel dimensions shrink, the likelihood of light intrusion into the MN increases. While increasing the depth of the DTI is effective in mitigating this issue, it alone is not sufficient.

**Figure 5:**
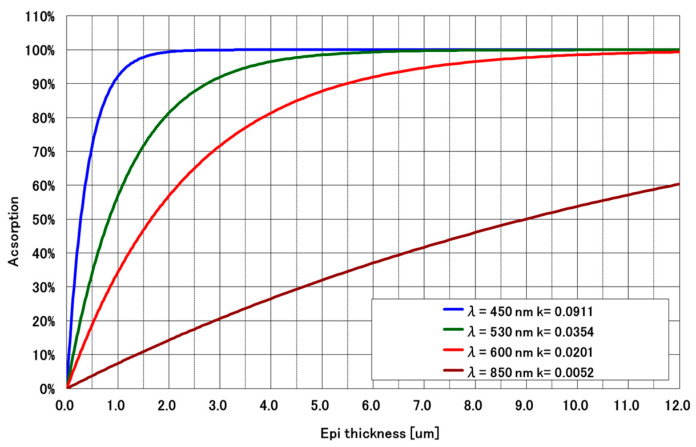
Relationship between epitaxial film thickness and absorption.

Since deepening the DTI alone was predicted to be insufficient for suppressing PLS in small pixels, we investigated the effect of increasing the epitaxial layer thickness. Figure 5 illustrates the relationship between epitaxial film thickness and optical absorption, based on simulation results [14]. When the epitaxial film thickness is 6 µm, blue light is completely absorbed by the silicon. Green light is also almost completely absorbed. However, 9% of red light is not absorbed. As can be seen from Figure 4, increasing the epitaxial thickness results in more absorption of red light. Therefore, we decided to increase the epi thickness in the newly developed BSI GS. However, there is a limit to how thick the film can be due to the actual manufacturing process. The epitaxial thickness was determined by the maximum thickness at which the alignment marks can be read during the fabrication process. The maximum film thickness was 8.5 μm. By increasing the epi thickness to 8.5 µm, only red light reaches the MN, and blue and green light do not reach the MN, resulting in a significant improvement in PLS. The combined use of a thick epitaxial layer and DTI is expected to significantly reduce PLS. However, when implementing deep DTI structures, careful consideration must be given to the signal readout from the PDs, as excessive trench depth may interfere with charge transfer efficiency.

**Figure 6:**
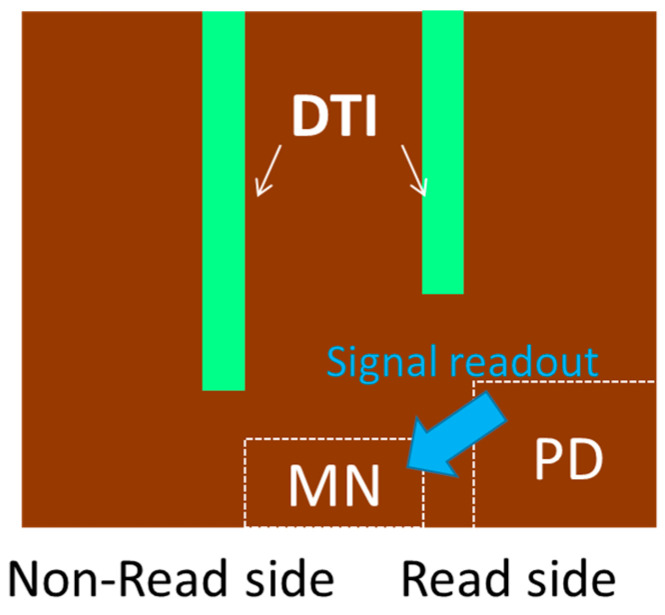
Dual-depth DTI structure.

Figure 6 shows the bottom shape of the DTI of the newly developed BSI GS. The DTI depth is different between the readout side and the non-readout side of the MN. Two primary factors determine the depth of DTI on the readout side. First, a gap of approximately 1.5 µm is necessary to enable signal readout from the PD to the MN. Second, it is essential to suppress electron diffusion current within the p-type well from reaching the memory node.

**Figure 7:**
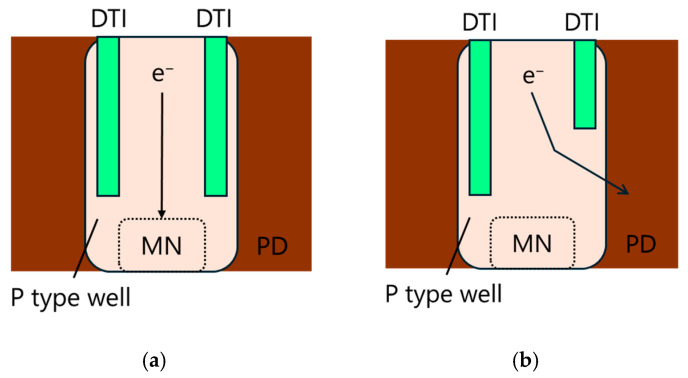
Schematics illustrating diffusion current flow of electrons in the p-type well surrounding the memory node, comparing (**a**) deeper DTI and (**b**) shallower DTI configurations on the readout side.

Figure 7 illustrates the expected electron diffusion current flow in the p-type well surrounding the memory node. When a deeper DTI is implemented on the readout side, photo-generated electrons located between the two DTIs are likely to diffuse toward the memory node due to the absence of alternative electron pathway within the p-type well. This diffusion current contributes as an additional source of PLS. In contrast, a shallower DTI on the readout side allows these electrons to be drained into the photodiode, thereby reducing PLS. The DTI on the non-readout side should be as deep as possible to prevent light from penetrating into the MN, so a dual-depth DTI structure was adopted.

**Figure 8:**
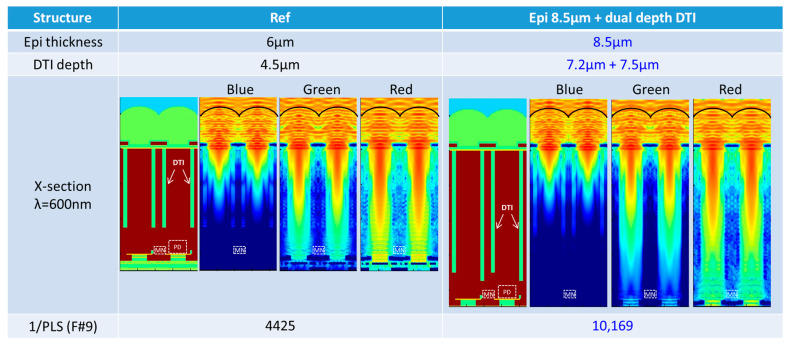
Simulation results and developed structure.

Figure 8 shows the results of optical simulations using the 3D-FDTD (finite-difference time domain) method, highlighting the effects of a thicker epitaxial layer and deeper DTI. Ansys Lumerical version 2021 R1.4 was used as the optical simulator software. The mesh size was set to 0.02 µm. The deep DTI effectively blocks light from reaching the MN, while a thicker epitaxial layer reduces light penetration. A 6 µm epitaxial thickness allows green and red light to reach the MN, making PLS reduction difficult. However, with an 8.5 µm epitaxial thickness, green light is entirely blocked and red light intensity is significantly reduced. This ensures low PLS under short-wavelength light sources used in inspection and measurement applications. Next, the optimal DTI depth was determined through TCAD simulations, indicating that an epitaxial thickness of 8.5 µm and DTI depths of 7.2 µm and 7.5 µm would achieve a 1/PLS of over 10,000. 1/PLS is calculated assuming a white halogen lamp.

### 3.2. Process Design

**Figure 9:**
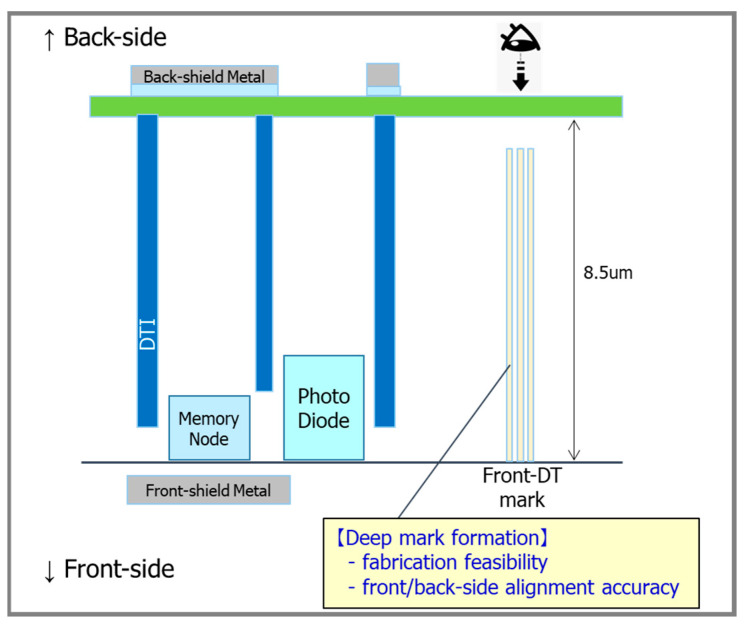
Key process structure of the dual-depth DTI and the alignment mark fabrication.

The main process challenges involved fabricating the thick epitaxial layer and the deep dual-depth DTI structure [15]. For the first time, we employed an 8.5 µm-thick epitaxial layer in a BSI GS pixel, comparable to the thickest commercial BSI GS products. We newly optimized the wafer thinning process down to 8.5 µm epitaxial layer which was made of the new starting material. In accordance with the thick-epi process, alignment mark was also optimized to meet the requirement of process feasibility and alignment accuracy for both of front-side and back-side as shown in Figure 9. Mark fabrication process has evolved from that of the conventional BSI. The dedicated mark process comprises the deep etching and deep-trench filling. The dual-depth DTI structure (7.2 µm and 7.5 µm) was achieved with a cost-effective, single-mask process. Specifically, dual-depth DTI is achieved by changing the mask size for shallow and deep DTI. The DTI layout, including its length and width, were carefully optimized and the actual DTI depth was successfully realized to meet the desirable depth above by using single etching process.

Although DTI sidewalls are known sources of photodiode dark current, which is typically proportional to DTI depth, we successfully suppressed dark current by optimizing the DTI formation process, which includes DTI etching condition, surface treatment process and the film formation process for passivation and filling within the DTI.

## 4. Results

**Figure 10:**
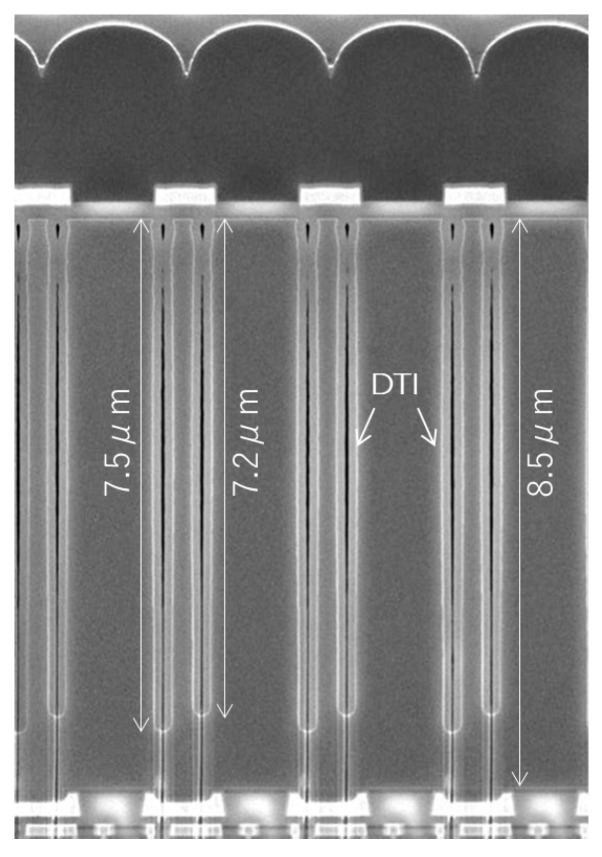
Cross-section of the dual-depth DTI.

Figure 10 presents the cross-section of the newly developed BSI GS sensor, fabricated using a 65 nm stacked BSI process with three Cu layers on the sensor wafer. The superior performance of the dual-depth DTI and thick epitaxial substrate was confirmed.

**Figure 11:**
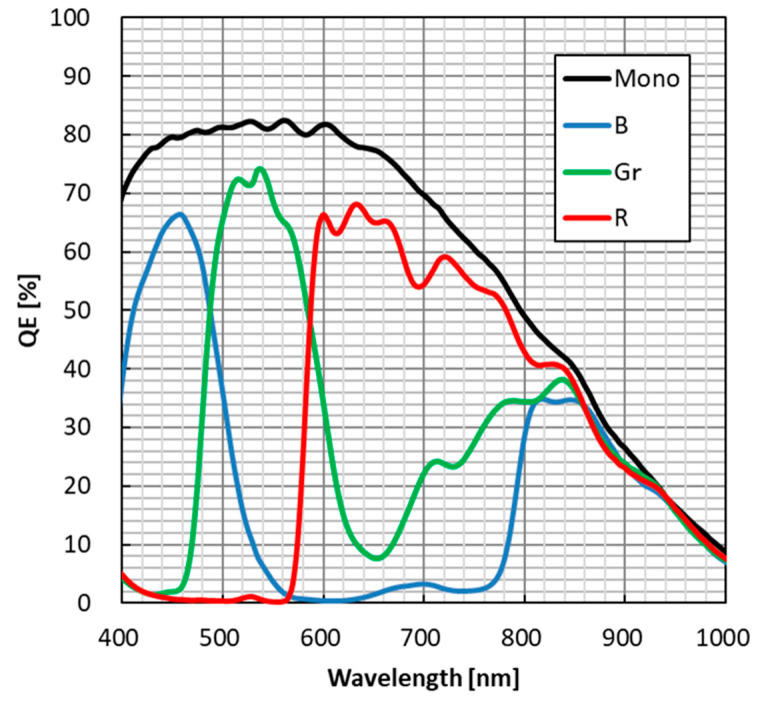
QE curve (color and mono).

Figure 11 shows QE curves for both color and monochrome samples. The QE for the green pixel (530 nm) reached 73%, while the QE for blue (450 nm) and red (600 nm) pixels were 66% and 68%, respectively. The peak QE of the monochrome sample was 83% at 550 nm. Despite the small pixel size, a high QE was achieved.

**Figure 12:**
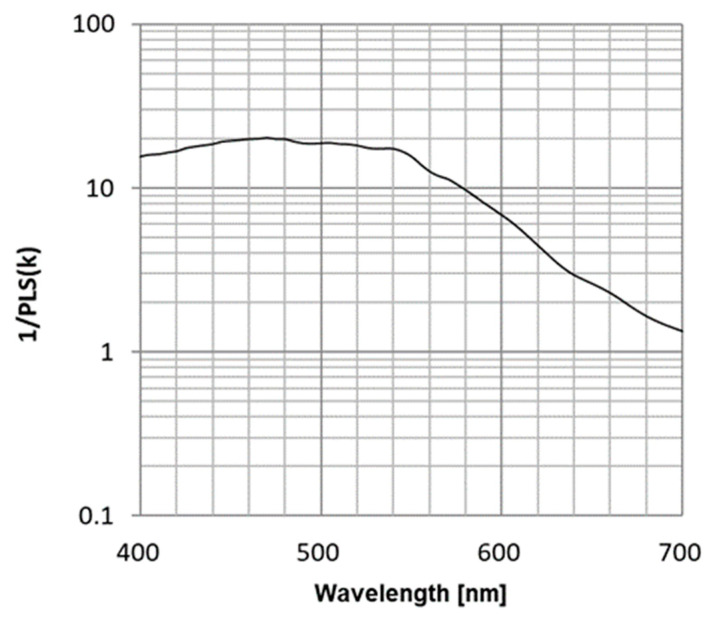
Wavelength dependence of 1/PLS (Mono).

Figure 12 presents the wavelength dependence of 1/PLS, demonstrating values exceeding 10,000 for wavelengths of 570 nm or less. This means that devices that use blue or green as a light source do not need to worry about PLS.

**Figure 13:**
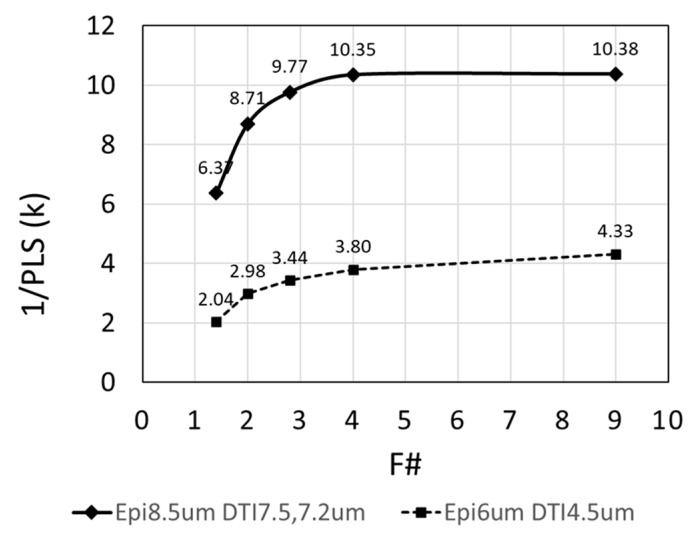
F#dependence of 1/PLS (Mono).

Figure 13 illustrates the F#-dependence of 1/PLS, showing that the monochrome sample achieved 1/PLS of 10,380 (−80.3 dB) at F# = 9, while a sample with a 6 µm epitaxial thickness had a lower 1/PLS of 4300. A white halogen lamp was used for the measurements. These values are very close to the results of optical simulations. The thick epitaxial and dual-depth DTI structures suppress the deterioration of PLS even when the F# is smaller.

**Figure 14:**
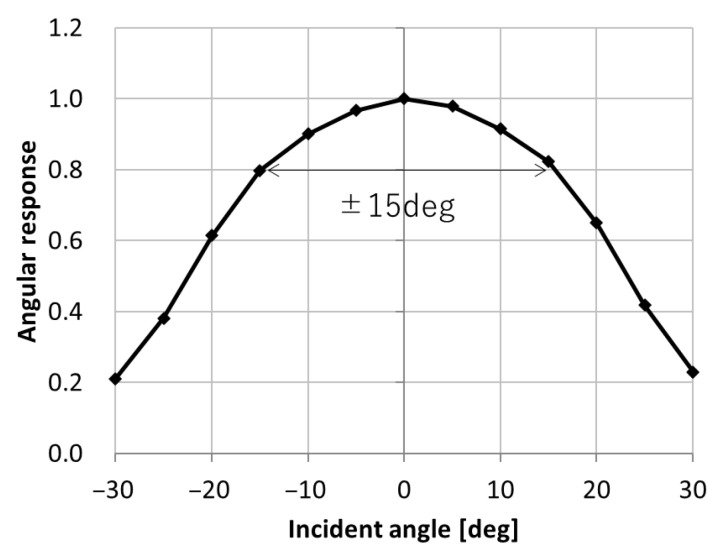
Angular response of QE (wavelength = 530 nm).

Figure 14 shows the angular response of QE. The measurement wavelength was 530 nm. Despite the small pixel size, QE remains above 80% of the peak value within a range of ±15 degrees.

**Figure 15:**
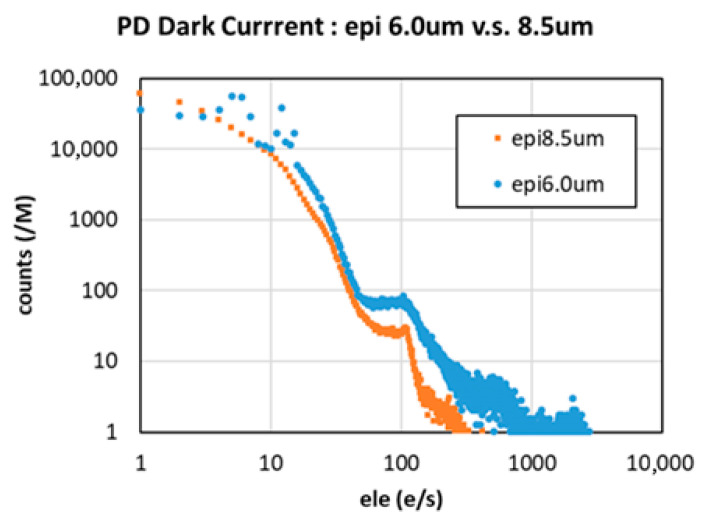
Comparison of dark current.

Figure 15 compares dark current levels between a 6 µm epi process and the new thicker-epi, deeper-DTI process. By optimizing DTI etching and buried material, dark current was minimized despite an increased DTI depth.

Table 1 summarizes the pixel performance, highlighting a noise level of 0.6 e^−^ due to the charge-domain GS-CDS architecture. The pixel array MTF is at least 40% at Nyquist frequency, making this a best-in-class, high-resolution, low-noise global shutter sensor.

## 5. Conclusions

We developed the world’s smallest charge-domain 2.2 µm BSI GS pixel, suitable for high-resolution machine vision sensors and consumer applications. To mitigate 1/PLS degradation from pixel miniaturization, we introduced a thicker epitaxial layer and a dual-depth DTI structure. A novel thick-film epi process was developed, achieving a peak QE of 83% (monochrome). The pixel maintains 80% of its peak QE at ±15 degrees.

The 1/PLS achieved 10,380 at F#9, surpassing the target threshold of 10,000. Even at F#2.8, 1/PLS remained high at 9770 (79.8 dB).

## Figures and Tables

**Table 1:** Pixel performance.

Process Technology	65 nm CIS BSI
Pixel Size	2.2 μm × 2.2 μm
Peak QE (Mono)	83% (wavelength = 560 nm)
1/PLS (F#9, white halogen light, Mono)	10,380
Angular response (80%)	>15 degrees
MTF @Nyquist frequency	>40% (wavelength = 520 nm)
Linear Full Well Capacity	5400 ele
Pixel noise @SF out (25 deg⋅C)	0.6 ele

## Data Availability

The data presented in this study are openly available on the IISW 2025 website https://imagesensors.org/papers/10.60928/k4oc-bw0e/ (accessed on 12 November 2025) [7].
